# Supplementary material for: Selective Hepatic Cbs Knockout Aggravates Liver Damage, Endothelial Dysfunction and ROS Stress in Mice Fed a Western Diet
Source: Int J Mol Sci. 2023 Apr 10;24(8):7019. doi: 10.3390/ijms24087019 (PMC10138434; doi:10.3390/ijms24087019)
Supplement: Supplementary file 1 [file ijms-24-07019-s001.zip › ijms-2312798-supplementary.pdf]

Table S1. Amino acid composition liver.

amino acids nmol/g liver	con	LiCKD
**Taurine**	6335 ± 348	6443 ± 476
**Aspartic acid**	2412 ± 877	2187 ± 354
**Threonine**	1084 ± 239	1270 ± 172*
**Serine**	1715 ± 353	1962 ± 280*
**Asparagine**	752 ± 190	859 ± 116
**Glutamic acid**	1096 ± 490	1107 ± 260
**Glutamine**	3644 ± 768	4276 ± 908
**Proline**	491 ± 137	597 ± 96*
**Glycine**	3711 ± 295	4068 ± 500*
**Alanine**	7147 ± 957	7777 ± 682*
**Citrulline**	292 ± 171	246 ± 42
**a-a-butyric acid**	96 ± 37	104 ± 29
**Valine**	1030 ± 199	1145 ± 146
**Methionine**	376 ± 90	456 ± 76*
**l-cystathionine**	47 ± 19	<LLOD*
**iso-leucine**	582 ± 124	641 ± 89
**leucine**	1625 ± 312	1775 ± 209
**Tyrosine**	747 ± 141	800 ± 115
**b-alanine**	163 ± 53	190 ± 63
**Phenylalanine**	758 ± 152	850 ± 110
**Ornithine**	1191 ± 270	1284 ± 177
**Lysine**	1574 ± 300	1659 ± 188
**Histidine**	933 ± 133	932 ± 91
**Tryptophan**	132 ± 74	117 ± 58
**Arginine**	26 ± 14	27 ± 11

* p<0.05 LiCKO over con.

Table S2. Body composition.

	12 weeks				20 weeks			
	con ND	LiCKO ND	con HFC	LiCKO HFC	con ND	LiCKO ND	con HFC	LiCKO HFC
Fat (g)	4.5 ± 0.6	4.8 ± 1.9*	14.6 ± 5.4†	14.4 ± 4.6†	4.5 ± 0.9	7.9 ± 2.5	19.7 ± 6.3†	18.4 ± 9.6†
Free fluid (g)	1.8 ± 0.2	2.2 ± 0.4	3.8 ± 1.1†	3.8 ± 0.8†	2.1 ± 0.2	2.6 ± 0.5	3.7 ± 0.8†	4 ± 1†
Lean (g)	21.2 ± 0.4	22.6 ± 0.6*	20 ± 1.3†	20.7 ± 1.4†	21 ± 2	22.2 ± 0.8	17.1 ± 3.7†	19.4 ± 4.4†
Fat (%)	16.3 ± 1.7	15.9 ± 4.9	37 ± 7.2†	36.2 ± 5.8†	16.4 ± 2.6	23.8 ± 5.1*	47.9 ± 10.6†	41.9 ± 13.9
Free fluid (%)	6.6 ± 0.7	7.5 ± 0.8	9.7 ± 0.9†	9.8 ± 0.8†	7.7 ± 1	8 ± 0.6	9.2 ± 1.2†	9.8 ± 2.7†
Lean (%)	77.1 ± 2.3	76.6 ± 5.5	53.2 ± 7.9†	54 ± 6.2†	75.9 ± 2.8	68.2 ± 5.7*	42.9 ± 9.6†	48.3 ± 11.9

Free fluid; plasma, cerebrospinal fluid and urine. *$p<0.05$ difference between con and LiCKO on same diet. † $p<0.05$ difference between ND and HFC for con or LiCKO.

Table S3. Pathology scores.

	12 weeks				20 weeks			
	con ND	LiCKO ND	con HFC	LiCKO HFC	con ND	LiCKO ND	con HFC	LiCKO HFC
Steatosis	0.0 ± 0.0	0.4 ± 0.5	1.4 ± 1.1†	1.8 ± 1.2†	0.0 ± 0.0	0.3 ± 0.7	1.3 ± 0.8†	2.6 ± 0.8*†
Ballooning	0.0 ± 0.0	0.0 ± 0.0	0.1 ± 0.4	0.3 ± 0.5	0.0 ± 0.0	0.0 ± 0.0	0.5 ± 0.5	0.1 ± 0.4
Loublar inflammation	0.4 ± 0.5	1.0 ± 0.0*	0.8 ± 0.5	1.7 ± 0.9*†	1.6 ± 0.5	0.7 ± 0.5*	1.5 ± 0.5	2.4 ± 0.8*†
NAS	0.4 ± 0.5	1.4 ± 0.5	2.3 ± 1.3†	3.8 ± 2.3†	1.6 ± 0.5	1.0 ± 0.9	3.3 ± 1.4†	5.1 ± 1.4*†

NAS; NAFLD activity score, *p<0.05 difference between con and LiCKO on same diet. † p<0.05 difference between ND and HFC for con or LiCKO.

Table S4. Acyl-carnitines in liver.

	12 weeks of diet				20 weeks of diet			
	con ND	LiCKO ND	con HFC	LiCKO HFC	con ND	LiCKO ND	con HFC	LiCKO HFC
total	136.0 ± 48.1	113.0 ± 49.6	124.6 ± 35.1	**37.3 ± 15.0*†**	206.6 ± 146.3	**52.0 ± 23.5***	108.8 ± 32.1	**36.2 ± 30.6***
C0	83.7 ± 27.8	81.9 ± 37.7	83.9 ± 24.3	**27.1 ± 11.7*†**	154.0 ± 110.9	**38.8 ± 19.4***	83.8 ± 23.1	**26.3 ± 21.0***
C2	38.3 ± 17.8	**19.3 ± 9.3***	23.4 ± 8.5	**4.9 ± 1.7*†**	32.6 ± 23.7	**7.6 ± 4.6***	9.6 ± 5.5	5.6 ± 6.2
C3	2.5 ± 1.1	**0.9 ± 0.4***	2.3 ± 0.9	**0.5 ± 0.3*†**	2.1 ± 2.0	**0.2 ± 0.1***	0.9 ± 0.5	**0.3 ± 0.2***
C4	0.31 ± 0.15	**0.15 ± 0.07***	0.13 ± 0.09†	**0.01 ± 0.03*†**	0.12 ± 0.10	**0.03 ± 0.04***	0.08 ± 0.05	0.04 ± 0.10*
C5:1	0.00 ± 0.00	0.00 ± 0.00	0.00 ± 0.00	0.00 ± 0.00	0.00 ± 0.00	0.01 ± 0.03	0.00 ± 0.00	0.00 ± 0.00
C5	0.20 ± 0.08	**0.13 ± 0.04***	0.21 ± 0.10	**0.05 ± 0.03*†**	0.19 ± 0.06	**0.06 ± 0.03***	0.12 ± 0.05	**0.04 ± 0.06***
C6	0.00 ± 0.00	0.00 ± 0.00	0.00 ± 0.00	0.00 ± 0.00	0.00 ± 0.00	0.00 ± 0.00	0.00 ± 0.00	0.00 ± 0.00
C8	0.00 ± 0.00	0.01 ± 0.02	0.14 ± 0.09†	**0.04 ± 0.04***	0.01 ± 0.03	0.00 ± 0.00	0.11 ± 0.03†	**0.01 ± 0.03***
C10:1	0.00 ± 0.00	0.01 ± 0.02	0.04 ± 0.04†	**0.00 ± 0.00***	0.00 ± 0.00	0.00 ± 0.00	0.06 ± 0.03†	**0.01 ± 0.02***
C10	0.03 ± 0.04	0.01 ± 0.02	0.04 ± 0.04	**0.00 ± 0.00***	0.03 ± 0.04	**0.00 ± 0.00***	0.04 ± 0.04	**0.01 ± 0.02***
C12:1	0.00 ± 0.00	0.04 ± 0.09	0.00 ± 0.00	0.00 ± 0.00	0.05 ± 0.12	0.00 ± 0.00	0.03 ± 0.04	**0.00 ± 0.00***
C12	0.00 ± 0.00	**0.05 ± 0.03***	0.00 ± 0.00	0.00 ± 0.00†	0.00 ± 0.00	0.00 ± 0.00	0.00 ± 0.00	0.00 ± 0.00
C14:1	0.00 ± 0.00	0.01 ± 0.02	0.00 ± 0.00	0.00 ± 0.00	0.03 ± 0.04	**0.00 ± 0.00***	0.01 ± 0.03	0.02 ± 0.03
C14	0.00 ± 0.00	0.03 ± 0.04	0.00 ± 0.00	0.00 ± 0.00†	0.00 ± 0.00	0.00 ± 0.00	0.01 ± 0.03	0.00 ± 0.00
C16:1	0.00 ± 0.00	0.01 ± 0.02	0.00 ± 0.00	0.00 ± 0.00	0.00 ± 0.00	0.00 ± 0.00	0.00 ± 0.00	0.00 ± 0.00
C16	0.01 ± 0.03	0.01 ± 0.03	0.06 ± 0.04†	**0.01 ± 0.03***	0.01 ± 0.03	0.00 ± 0.00	0.04 ± 0.04	**0.01 ± 0.03***
C18:2	0.01 ± 0.03	0.00 ± 0.00	0.02 ± 0.03	0.00 ± 0.00	0.00 ± 0.00	0.00 ± 0.00	0.00 ± 0.00	**0.00 ± 0.00**
C18:1	0.01 ± 0.03	0.03 ± 0.04	0.04 ± 0.04	**0.00 ± 0.00*†**	0.01 ± 0.03	0.00 ± 0.00	0.01 ± 0.03	0.01 ± 0.02
C18	0.00 ± 0.00	0.01 ± 0.02	0.03 ± 0.05	0.02 ± 0.03	0.01 ± 0.03	0.00 ± 0.00	0.01 ± 0.03	0.01 ± 0.03
C4OH+C3DC	4.27 ± 1.34	3.34 ± 1.86	3.59 ± 1.15	**1.14 ± 0.55*†**	5.92 ± 2.47	**1.95 ± 1.01***	3.07 ± 1.66	1.08 ± 0.94
C5OH+C4DC	0.60 ± 0.20	0.78 ± 0.43	0.88 ± 0.28†	**0.25 ± 0.14*†**	0.83 ± 0.63	**0.30 ± 0.15***	0.56 ± 0.17	**0.21 ± 0.24***
C5DC	5.32 ± 1.62	4.99 ± 2.11	8.71 ± 3.50†	**2.62 ± 1.42*†**	8.80 ± 7.54	**2.47 ± 1.01***	8.49 ± 4.14	**2.00 ± 1.72***
C6DC	0.68 ± 0.24	**0.45 ± 0.20***	0.53 ± 0.19	**0.27 ± 0.11*†**	0.69 ± 0.49	**0.31 ± 0.15***	0.92 ± 0.45	**0.27 ± 0.16***
C12OH	0.34 ± 0.11	0.86 ± 1.27	0.29 ± 0.13	0.29 ± 0.11	1.35 ± 2.23	0.29 ± 0.05	0.56 ± 0.44	0.22 ± 0.09†

Units: nmol/g liver. *P<0.05 difference between con and LiCKO on same diet (also indicated in bold), † P<0.05 difference between ND and HFC for con or LiCKO, ns=non-significant.

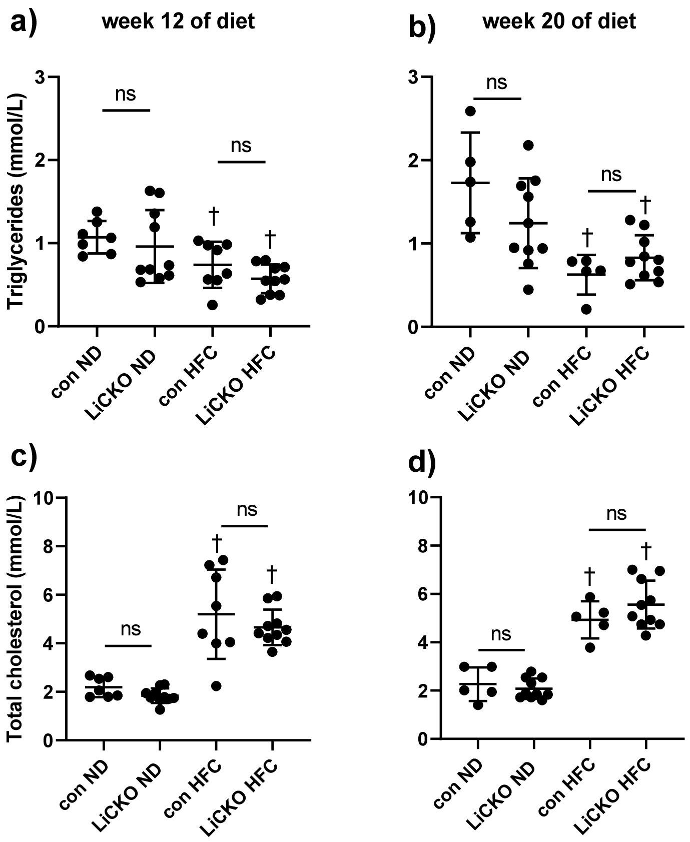

Figure S1. Dyslipidemia is not affected in LiCKO. (a,b) Plasma triglycerides at week 12 and 20, respectively; (c,d) Plasma total cholesterol at week 12 and 20, respectively. *P<0.05 difference between con and LiCKO on same diet, † P<0.05 difference between ND and HFC for con or LiCKO, ns=non-significant.

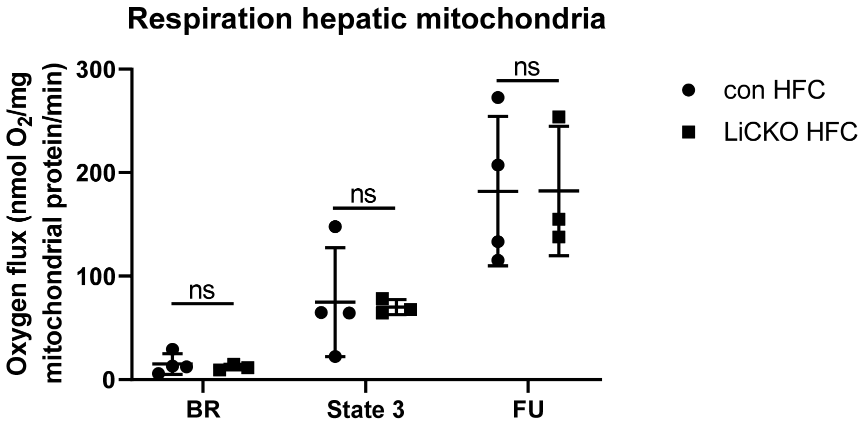

Figure S2. Respiration in isolated liver mitochondria using palmitoyl-carnitine is not affected in LiCKO. ns; non-significant, BR; basal respiration, FU; full uncoupling.

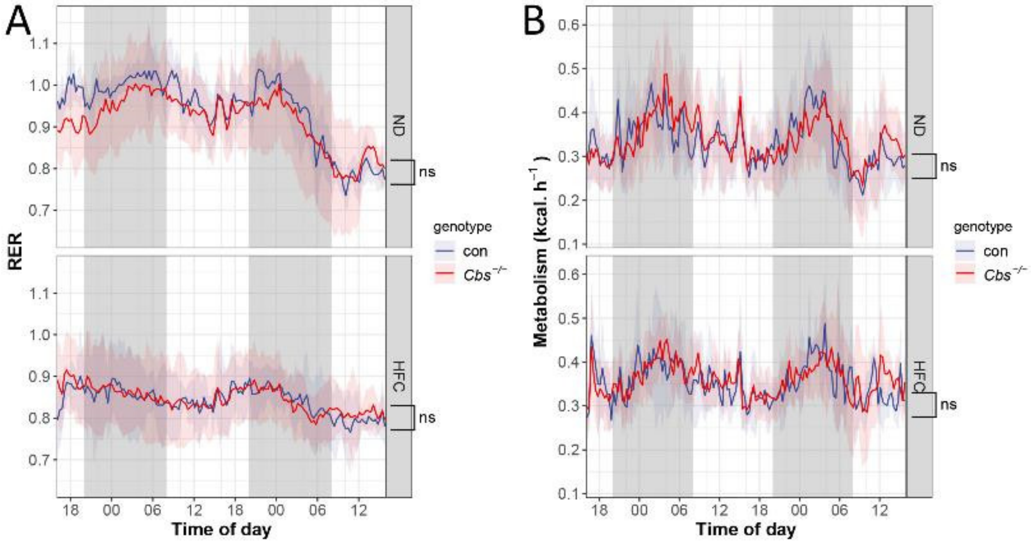

Figure S3. Respiratory exchange ratio and metabolism is not affected in LiCKO. ns=non-significant.
